# DOES THE DROP IN PORTAL PRESSURE AFTER ESOPHAGOGASTRIC DEVASCULARIZATION AND SPLENECTOMY INFLUENCE THE VARIATION OF VARICEAL CALIBERS AND THE REBLEEDING RATES IN SCHISTOSOMIASIS IN LATE FOLLOW-UP?

**DOI:** 10.1590/0102-672020210002e1581

**Published:** 2021-10-18

**Authors:** Walter de Biase SILVA-NETO, Claudemiro QUIRESE, Eduardo Guimarães Horneaux de MOURA, Fabricio Ferreira COELHO, Paulo HERMAN

**Affiliations:** 1 Serviço de Cirurgia Geral e Aparelho Digestivo, Departamento de Clínica Cirúrgica, Faculdade de Medicina, Universidade Federal de Goiás, Goiânia, GO, Brasil; 2 Serviço de Endoscopia, Hospital das Clínicas e Departamento de Gastroenterologia, Faculdade de Medicina, Universidade de São Paulo, São Paulo, SP, Brasil; 3 Serviço de Cirurgia do Fígado, Hospital das Clínicas e Departamento de Gastroenterologia, Faculdade de Medicina, Universidade de São Paulo, São Paulo, SP, Brasil

**Keywords:** Schistosomiasis mansoni, Portal hypertension, Surgery, Portal pressure, Esophageal and gastric varices, Esquistossomose mansoni, Hipertensão portal, Cirurgia, Pressão na veia porta, Varizes esofágicas e gástricas

## Abstract

***Background*:**

The treatment of choice for patients with schistosomiasis with previous episode of varices is bleeding esophagogastric devascularization and splenectomy (EGDS) in association with postoperative endoscopic therapy. However, studies have shown varices recurrence especially after long-term follow-up.

***Aim*:**

To assess the impact on behavior of esophageal varices and bleeding recurrence after post-operative endoscopic treatment of patients submitted to EGDS.

***Methods*:**

Thirty-six patients submitted to EGDS were followed for more than five years. They were divided into two groups, according to the portal pressure drop, more or less than 30%, and compared with the behavior of esophageal varices and the rate of bleeding recurrence.

***Results*:**

A significant reduction on the early and late post-operative varices caliber when compared the pre-operative data was observed despite an increase in diameter during follow-up that was controlled by endoscopic therapy.

***Conclusion*:**

The drop in portal pressure did not significantly influence the variation of variceal calibers when comparing pre-operative and early or late post-operative diameters. The comparison between the portal pressure drop and the rebleeding rates was also not significant.

## RESUMO

***Racional*:**

O tratamento de escolha para pacientes com hipertensão portal esquistossomótica com sangramento de varizes é a desconexão ázigo-portal mais esplenectomia (DAPE) associada à terapia endoscópica. Porém, estudos mostram aumento do calibre das varizes em alguns pacientes durante o seguimento em longo prazo.

***Objetivo*:**

Avaliar o impacto da DAPE e tratamento endoscópico pós-operatório no comportamento das varizes esofágicas e recidiva hemorrágica, de pacientes esquistossomóticos. Métodos: Foram estudados 36 pacientes com seguimento superior a cinco anos, distribuídos em dois grupos: queda da pressão portal abaixo de 30% e acima de 30% comparados com o calibre das varizes esofágicas no pós-operatório precoce e tardio além do índice de recidiva hemorrágica.

Após a DAPE houve diminuição significativa no calibre das varizes esofágicas que, durante o seguimento aumentaram de calibre e foram controladas com tratamento endoscópico. A queda da pressão portal não influenciou significativamente o comportamento do calibre das varizes no pós-operatório precoce nem tardio nem os índices de recidiva hemorrágica.

***Conclusão*:**

A queda na pressão portal não influenciou significativamente a variação dos calibres das varizes ao comparar os diâmetros pré e pós-operatórios precoces ou tardios. A comparação entre a queda de pressão do portal e as taxas de ressangramento também não foi significativa.

## INTRODUCTION

Mansonic schistosomiasis, in its hepatosplenic form is characterized by an obstruction of portal flow, caused by fibrosis in the periportal space. This obstruction, along with increased portal flow secondary to splenic vein drainage, represent the disease’s main physiopathologic mechanism. Increased portal flow is supported by the hyperdynamic systemic state, caused by increased cardiac output and by decreased peripheral vascular resistance. In these patients, the spleen acts as a large arterial-venous fistula, with blood flow being diverted from systemic to portal circulation^3^ ^,^ ^19^.

The rupture of esophageal varices and the consequent gastrointestinal hemorrhage is the main cause of the high mortality rate of this disease^8^. It is estimated that 30-40% of hepatosplenic form patients will present with upper gastrointestinal bleeding due to portal hypertension, with mortality rates ranging between 11.7% and 20%. Additionally, the risk of hemorrhagic recurrence in the first year is about 70%^13^ ^,^ ^14^. 

The treatment of choice for these patients, therefore, is surgery, and the most recommended procedure is esophagogastric devascularization and splenectomy (EGDS). The technique follows well-established standards and presents favorable results and safety in avoiding post-operative hepatic encephalopathy, a common complication of portal-systemic shunts. EGDS, on the other hand, present bleeding recurrence rates ranging from 6-29%^1^ ^,^ ^2^ ^,^ ^5^ ^,^ ^6^ ^,^ ^8^ ^,^ ^10^ ^,^ ^12^ ^,^ ^20^. For this reason, post-operative endoscopic treatment of esophageal varices is routinely performed^10^ ^,^ ^15^ ^,^ ^18^ ^,^ ^23^.

Sakai et al. (1990)^21^ demonstrated that endoscopic sclerotherapy following prior splenectomy or EGDS was more efficient than sclerotherapy alone in treating variceal bleeding, lowering rebleeding rates to under 7%. Makdissi et al. ^15^ showed that, in the long term, 57% of patients whose esophageal varices had been eliminated after EGDS with post-operative endoscopic treatment, presented with recurrence. Therefore, continuous endoscopic monitoring and treatment are recommended.

Studies combining esophagogastric devascularization and post-operative endoscopic therapy are scarce, and most use sclerotherapy as the endoscopic therapy, instead of elastic band ligation. On the other hand, studies that assess the progress of patients submitted to endoscopic elastic band ligation to treat esophageal varices involve mainly cirrhotic patients. Therefore, the efficacy of this technique in the post-operative treatment of esophageal varices and the rate of bleeding recurrence in patients with schistosomiasis who were submitted to EGDS remains an unanswered question.

The aim of this study was to assess the long-term impact on the behavior of esophageal varices and their hemorrhagic recurrence in the post-operative endoscopic treatment of patients who presented with schistosomiasis and were submitted to EGDS, in two Brazilian university hospitals.

## METHODS

This study was approved by the Ethics Committees of both the Clinics Hospital of the University of São Paulo, and the Clinics Hospital of the Federal University of Goiás, Brazil. It was retrospective investigating 176 patients who presented with portal hypertension due to hepatosplenic schistosomiasis with prior bleeding from esophageal variceal rupture, and who were submitted to esophagogastric devascularization and splenectomy at the University of São Paulo School of Medicine, and at the Goiás Federal University School of Medicine. From that group, 59 had been subjected to a protocol to measure the intra-operative portal pressure before and immediately after the surgery, with the goal of evaluating the impact in portal pressure due to the procedure. Of them, 36 had complete medical records, and were monitored for more than five years after the surgery. 

The inclusion criteria were: over 18 years old; had had a previous episode of upper gastrointestinal bleeding due to esophageal variceal rupture and submitted to EGDS with monitoring of the intra-operative portal pressure; had been diagnosed with mansonic schistosomiasis based on epidemiology and clinical evidence, and confirmed by histopathologic examination with a biopsy during the surgery; had post-operative monitoring (clinical and endoscopic) for more than five years.

The exclusion criteria were: chronic alcoholism or positive tests for hepatitis B or C; clinical or laboratory evidence of hepatocellular insufficiency, or histopathologic evidence of other hepatopathies; and portal vein thrombosis.

The pre- and post-operative endoscopic evaluations, and the operative technique with the portal pressure evaluation have been previously described^24^.

The endoscopic data for the caliber of esophageal varices of 21 patients in the early post-operative period (the first 30 days) were evaluated and compared with the respective calibers in the late post-operative period (greater than five years).

Patients were divided into two groups, according to the portal pressure drop, in more or less than 30%.The threshold value of 30% was chosen because it was the average indicated by studies that measured the portal pressure before and after EGDS or splenectomy^4^ ^,^ ^7^ ^,^ ^11^ ^,^ ^22^.

The following evaluations were done based on analyzed data: variceal calibers in the pre-operative compared to early and late post-operative periods; drop of portal pressure and the behavior of the varices in the early and late post-operative period; and drop of portal pressure and the rate of post-operative bleeding recurrence.

### Statistical analysis

The Kolmogorov test was used to analyze the caliber of the varices in the pre-operative, early post-operative, and late post-operative periods. The exact Fisher test was used to compare the drop in portal pressure with bleeding recurrence. All analyses considered a significance level of 5%.

## RESULTS

Average patients age was 35 years (22-49), 52.7% were male and 47.3% female.

All 36 patients had pre-operative esophageal varices of medium or large caliber. Eighteen (50%) had grade IV varices; 16 (44.4%) grade III, and two (5.4%) grade II.

Regarding the evaluation of the drop in portal pressure 21 (58.3%) drop less than 30%, while (41.7%) greater than 30%.

**FIGURE 1:**
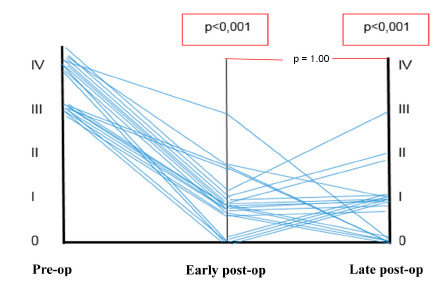
Evolution of varices caliber in the pre-operative and early and late post-operative periods

When the calibers of pre-operative varices were compared with those of the 21 patients for whom early post-operative data was available, a significant reduction in diameter was observed (p<0.001, Table 1, Figure 1).

When variceal calibers in the pre-operative period were compared with those in the late post-operative period, a significant reduction in diameter was found (p<0.001, Table 2, Figure 1).

**TABLE 1:** Comparison of variceal diameter in the pre-operative and early post-operative periods

Variceal caliber	Pre-operative		Early post-operative		p
	n	%	n	%	
0	-	0.0	4	19.0	
I	-	0.0	13	61.9	
II	-	0.0	3	14.3	< 0.001
III	10*	44.4	1	4.8	
IV	11*	50.0	-	0.0	
Total	21	100.0	21	100.0	

*Pre-operative results for patients for whom early post-operative data was available Fisher test

**TABLE 2:** Comparison of variceal diameter in the pre-operative and late post-operative periods

Variceal diameter	Pre-operative		Late post-operative		p
	n	%	n	%	
0	-	0.00	14	38.9	
I	-	0.00	12	33.3	
II	2	5.6	7	19.4	<0.001
III	16	44.4	3	8.4	
IV	18	50.0	-	0.0	
Total	36	100	36	100	

Fisher test

Of the 21 patients who were evaluated in the early post-operative period and monitored in the post-operative period, 13 (61.9%) showed an increase in the number of varices during the monitoring.

No statistically significant difference was found when the variation in drop of portal pressure (above and below 30%) was analyzed against the variation of variceal caliber, in a comparison between pre- and early post-operative data. The same result was not found when comparing the caliber of the varices in the pre- and late post-operative periods.

When comparing the portal pressure drop (above and below 30%), in the group with a drop lower than 30%, 16 (76.2%) did not present bleeding recurrence, while five (23.8%) did.In the group with a drop higher than 30%, 14 (93.3%) did not present bleeding recurrence, while one (6.7%) did. The comparison between the two groups of portal pressure drop and their relations to the rates of bleeding recurrence was not significant (p=0.20, Table 3).

**TABLE 3:** Bleeding recurrence rate according to the drop in portal pressure

Drop in portal pressure (%)	Hemorrhagic recurrence				p
	No		Yes		
	n	%	n	%	
<30	16	76.2	5	23.8	
					0.20
>30	14	93.3	1	6.7	
TOTAL	30		6		

Fisher test

## DISCUSSION

This study found a significant reduction in the caliber of the varices evaluated endoscopically in the early post- in comparison with the pre-operative period; in other words, the surgical treatment per se had a positive influence in reducing the diameter of esophageal varices. However, the intra-operative variation in drop of portal pressure (greater than 30%) did not have a significant influence on the diameter of esophageal varices in the immediate post-operative period (no influence of endoscopic therapy).

The reduction in caliber of the esophageal varices was not significant when comparing the pre- and post-operative (early and late) periods in relation to the drop in portal pressure (above and below 30%), in absolute values. This supports the impression that the interruption of the circuit between the portal system and other venous systems, rather than the drop in portal pressure during surgery, is responsible for the positive results of EGDS. Although this significant reduction in variceal caliber in the early post-operative period was followed by a stabilization or reduction of variceal caliber in long term monitoring, a considerable number of patients showed increased variceal calibers during post-operative monitoring.

It is possible that, with time, the changes caused by the surgery gradually lose their effect; in this regard, we were able to observe that the increase in variceal caliber during endoscopic monitoring was independent of the drop in post-operative portal pressure. Another finding that supports this theory is that, when the bleeding recurrence rates were compared in the two patient groups with intra-operative portal pressure drops lower or greater than 30%, the difference was also not significant.

It is important to point out that in post-operative endoscopic monitoring, interventions on the varices were made whenever necessary, either through sclerotherapy or, more recently, through elastic band ligation. The recurrence or progression in variceal caliber evidenced the important role of post-operative endoscopic therapy. Reinforcing this concept, Makdissi et al. 15 demonstrated, in long term monitoring after EGDS, that in 56.6% of patients whose varices had been eliminated by surgery associated to endoscopic treatment, the varices recurred or increased in diameter during monitoring. On the other hand, when was assessed the caliber of the varices in the early post-operative period in comparison with the late one, was not found a significant difference; this seems to occur in the majority of patients due to the endoscopic therapy.

The effectiveness of EGDS associated with endoscopic therapy does not seem to be replicated by the use of either treatment on its own, as shown by Strauss et al. 25 when comparing surgery alone and surgery associated to endoscopic treatment for schistosomiasis-associated portal hypertension. The authors showed that, in patients subjected to EGDS alone (not complemented by other therapies), after an average monitoring period of six years, 28% of patients presented with varices of equal or larger caliber in comparison to the early post-operative period.

Lacet et al. 14 showed that, with an average monitoring period of six years, although a significant reduction in variceal caliber was observed in both, patients subjected to endoscopic therapy alone and the ones to endoscopy and EGDS, 8% of those patients with endoscopic therapy alone had a recurrence of large caliber varices.

The assessment of the hemodynamic (systemic and portal) impact of EGDS has been the object of research at both centers of this research since the 1990s. Most of the papers on the subject evaluated only the immediate results of surgery, without investigating the long-term impact of these interventions. The late results of associating EGDS with endoscopic treatment, regarding the caliber of esophageal varices and bleeding recurrence, are still scarcely studied15,23,24.

EGDS with endoscopic therapy has shown low rates of bleeding recurrence, between 5.1% and 15.7%9,14,15,21,22.In this work, the rate of bleeding recurrence by rupture of esophageal varices was 16.6%, the highest among all studies that performed similar analyses; however, this research present the longest post-operative period. The higher percentage of patients with bleeding recurrence supports the statement of Ferreira et al9. that the bleeding recurrence rate increases with longer post-operative follow-up. 

This study had a few limitations, such as a less than ideal sample size.That is because the number of patients with schistosomiasis has dropped significantly both in São Paulo and Goiás,due to a decrease in the occurrence, morbidity, and mortality of schistosomiasis in our country16,17.Another limitation is the fact that this is a retrospective study, which decreases its conclusive value; however, it has provided the chance to evaluate a very long monitoring period, which would not be possible in a prospective study.

Despite these inherent limitations, this study had the advantage of analyzing the drop in intra-operative portal pressure, a rare reported data (only four studies address this data for EGDS patients), correlating this drop to the behavior of esophageal varices and bleeding recurrence over a long post-operative period (10 years). Another benefit of this study is comparing therapeutic results in a cohort of patients in which was assessed the immediate influence of surgical treatment (measurement of portal pressure drop), as well as the short-term (prior to the influence of endoscopic therapy) and long term impacts of the procedure, in individuals that were not influenced by any other diseases or disorders, as observed in cirrhotic patients. 

The limited size of the sample does not allow us to infer what type of treatment - whether surgery with endoscopic therapy or endoscopic therapy alone - would be the best approach for these patients. Further studies are necessary to understand the role of surgery in the long-term control of esophageal varices in schistosomiasis patients.

## CONCLUSION

The drop in portal pressure did not significantly influence the variation of variceal calibers when comparing pre-operative and early or late post-operative diameters. The comparison between the portal pressure drop and the rebleeding rates was also not significant. 
